# fimpera: drastic improvement of Approximate Membership Query data-structures with counts

**DOI:** 10.1093/bioinformatics/btad305

**Published:** 2023-05-17

**Authors:** Lucas Robidou, Pierre Peterlongo

**Affiliations:** Univ. Rennes, Inria, CNRS, IRISA, Rennes, F-35000, France; Univ. Rennes, Inria, CNRS, IRISA, Rennes, F-35000, France

## Abstract

**Motivation:**

High throughput sequencing technologies generate massive amounts of biological sequence datasets as costs fall. One of the current algorithmic challenges for exploiting these data on a global scale consists in providing efficient query engines on these petabyte-scale datasets. Most methods indexing those datasets rely on indexing words of fixed length *k*, called *k*-mers. Many applications, such as metagenomics, require the abundance of indexed *k*-mers as well as their simple presence or absence, but no method scales up to petabyte-scaled datasets. This deficiency is primarily because storing abundance requires explicit storage of the *k*-mers in order to associate them with their counts. Using counting Approximate Membership Queries (cAMQ) data structures, such as counting Bloom filters, provides a way to index large amounts of *k*-mers with their abundance, but at the expense of a sensible false positive rate.

**Results:**

We propose a novel algorithm, called fimpera, that enables the improvement of any cAMQ performance. Applied to counting Bloom filters, our proposed algorithm reduces the false positive rate by two orders of magnitude and it improves the precision of the reported abundances. Alternatively, fimpera allows for the reduction of the size of a counting Bloom filter by two orders of magnitude while maintaining the same precision. fimpera does not introduce any memory overhead and may even reduces the query time.

**Availability and implementation:**

https://github.com/lrobidou/fimpera.

## 1 Introduction

Public data banks providing sequencing data or assembled genome sequences are growing at an exponential rate (Cummins et al. 2022), and faster than computational power. Searching for a sequence of interest among datasets is a fundamental need that enables for instance a better understanding of genetic changes in tumors, offering precious information about the diagnosis and treatment of cancer (Tomczak et al. 2015), or enabling to study at a large scale the distribution and adaptation of life in oceans (Sunagawa et al. 2020). However, no method scales to the dozens of petabytes of data already available today. Thus, new computational methods are required to perform a search against datasets.

Querying datasets can be done precisely by aligning genome sequences [e.g. using Blast-like (Altschul et al. 1990) algorithms]. However aligning sequences is computational-resources intensive and cannot be applied efficiently when datasets are raw sequencing data. Thus, queries on large-scale datasets are usually done through *k*-mers presence/absence: each dataset is represented by its set of *k*-mers, and a query is represented by its sequence of *k*-mers. The ratio of the *k*-mers in the query and in the dataset over all the *k*-mers in the query approximates the similarity between the query and the dataset.

The challenge is to index hundreds of billions of distinct *k*-mers across thousands of datasets. Methodological developments have thus been made to index every *k*-mer of a dataset. Some methods use Approximate Membership Query (AMQ) data structures, e.g. Bloom filters, to store the presence/absence of *k*-mers, as for instance SBT (Solomon and Kingsford 2016) or HowDeSBT (Harris and Medvedev 2020); see Marchet et al. (2021) and Chikhi et al. (2022) for a survey of the approaches. However, very few methods tackle the issue of recording the abundance of the indexed *k*-mers. The information about the abundance is however crucial for many biological applications such as in transcriptomics, metagenomics, or metatranscriptomics analyses. We can distinguish three strategies for indexing *k*-mer abundances:

- Explicitly storing couples (*k*-mer, abundances). This may be done simply using hash tables with *k*-mers as keys and their abundances as values. However, this approach cannot scale to dozens or billions of distinct *k*-mers. More compact solutions reduce the *k*-mer set size using assemblies such as compact de Bruijn graph representation [see Bowe et al. (2012) or Alanko et al. (2022)] or a spectrum-preserving string set (SPSS) (Rahman and Medvedev 2020). However, these approaches still require the explicit association of each represented *k*-mer to its set of abundances in each indexed dataset. Additionally, computing the de Bruijn graph or the SPSS of a set of reads requires intensive computational resources. As such, while being effective on, e.g. full genomes, it becomes inefficient when representing highly complex and diverse datasets such as metagenomic seawater for instance. Note that once the set of *k*-mer is computed, the memory cost of adding counts is not the bottleneck [see Pibiri (2022a), in which the storage of abundances requires only a fraction of the total memory usage]. Moreover, counts can be stored as an approximation [see Shibuya et al. (2022a)] or exactly, as described below.
- Using a minimal perfect hash function (MPHF) such as Limasset et al. (2017), or more recently Pibiri and Trani (2021). MPHFs enable the association of an indexed key to a specific and unique value. They provide an efficient way to associate a key to any piece of information. In our context, a *k*-mer can be associated with its abundance in various datasets. Not talking about their construction computation time, these MPHFs do not enable the detection of whether a queried *k*-mer belongs to the original indexed set. Hence, they provide erroneous information for any non-indexed *k*-mer, limiting their usage in this context.
  To circumvent this problem, certain MHPFs rely on an explicit representation of the indexed *k*-mer set and hence also fall into the previous strategy. This is for instance the case of Blight (Marchet et al. 2019) and SSHash (Pibiri 2022b). However, for the same reasons as previously mentioned, these approaches cannot be applied to highly complex and diverse datasets.
- Use an AMQ, adding the abundance information instead of only the presence/absence of each *k*-mer, in this case, we call this AMQ a “counting AMQ”. Using a counting AMQ, the count information cannot be stored using a distinct structure in which the redundant count information between *k*-mers could be compressed, as proposed in Shibuya et al. (2022b). Instead, in a counting AMQ, the abundance of each stored *k*-mer is explicitly represented and thus is not space-efficient. Adding abundance information in an AMQ at fixed memory usage increases its false positive rate. For example, BIGSI (Bradley et al. 2019) relies on Bloom filters with a high false positive rate, e.g. 25% false positive rate per *k*-mer query. At constant memory usage, adding the information of abundance using, e.g. five bits per cell would yield an extremely high false positive rate that could reach up to 70%, which is not tolerable.

In this paper, we propose a wrapper to improve any existing counting AMQ. The method we introduce is called fimpera. It generalises a previous contribution called findere (Robidou and Peterlongo 2021). In short, fimpera splits every *k*-mer into *s*-mers (with k≥s>0) and then associates the abundance of a *k*-mer with its constituent *s*-mers in a counting AMQ. This allows us to retrieve the abundance of a *k*-mer at query time via its *s*-mers count. We show that, when compared to the original counting AMQ indexing of kmers, fimpera improves abundance correctness while lowering the false positive rate by an order of magnitude without generating false-negative calls or underestimating the abundance of a kmer and without requiring additional time for query execution. Alternatively, for a fixed false positive rate value, the fimpera strategy allows reducing the size of the cAMQ by two orders of magnitude.

The fimpera algorithm can be used on top of any query made using any kind of counting AMQ. However, our implementation and tests are proposed on top of a counting Bloom Filter (cBF) that uses a unique hash function. This choice is motivated by the fact that cBFs are the simplest and are widespread data structures for dealing with billions of elements, and by the fact that state-of-the-art indexing tools based on counting AMQ [Cobs (Bingmann et al. 2019), HowDeSBT (Harris and Medvedev 2020)] impose the usage of a unique hash function.

Additionally, the fimpera algorithmic needs led us to propose a novel algorithm for computing the sliding window minima (resp. maxima): the minimal (resp. maximums) values of all sub-arrays of a fixed size over an array of *x* values. This algorithm runs in O(x) time and requires no dynamic memory allocation. This contribution may be useful outside the fimpera context. Its novelty is that being in place, it uses no additional memory, while other approaches use memory, i.e. linear with the size of the intervals. This makes it the fastest known algorithm to perform this task. It is available at https://github.com/lrobidou/sliding-minimum-windows along with a benchmark comparing it to other solutions.

The fimpera contribution is publicly available at https://github.com/lrobidou/fimpera.

## 2 Materials and methods

### 2.1 Background

A *k*-mer is a word of length *k* over an alphabet Σ. Given a sequence *S*, |S| denotes the length of *S*. In the current framework, we consider a dataset to be composed of a multiset of sequences. Given a dataset *D*, $D_{k}$ denotes the multiset of *k*-mers extracted from *D*.

The abundance of a *k*-mer *d* (the number of times *d* appears) in $D_{k}$ is represented by $\text{abundance}(D_{k},d)$. We consider that a *k*-mer is “present” in D if $\text{abundance}(D_{k},d)>0$, else ($\text{abundance}(D_{k},d)=0$) the *k*-mer is “absent”.

A counting AMQ data structure represents a multiset of elements $D_{k}$. It can be queried with any element *d*; the query’s response on a counting AMQ, denoted by *n*, is always either correct or overestimated, i.e. $n\geq \text{abundance}(D_{k},d)$. If $n=\text{abundance}(D_{k},d)$, the counting AMQ reports the correct abundance, otherwise it reports an overestimation. Note that underestimation is not possible.

In particular, if $\text{abundance}(D_{k},d)=0$ and *n *>* *0, then *d* is found in the counting AMQ even if it is absent from D. This particular case is a false positive call. The false positive rate of a counting AMQ, denoted by FPR_cAMQ_, is defined by ${\text{FPR}}_{\text{cAMQ}}=\frac{\#\text{FP}}{\#\text{FP}\;+\;\#\text{TN}}$ with #FP and #TN denoting respectively the number of false positive calls and the number of true negative calls (*n *=* *0). FPR_cAMQ_ depends on the used counting AMQ strategy and on the amount of space used by this counting AMQ.

**Figure 1.**
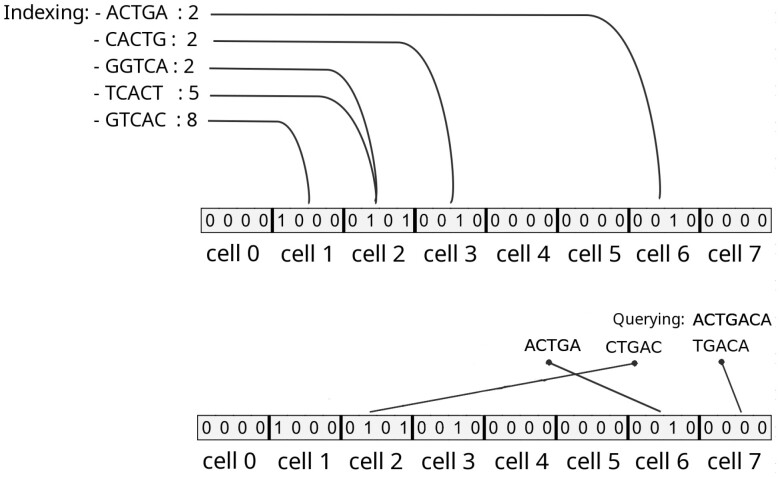
Toy example of a counting Bloom Filter using a unique hash function, with *k*-mers of length *k *=* *5, and *b *=* *4 bits per element. *Top, indexing*: each indexed element (*k*-mer) is hashed to a slot, and its abundance is stored in that slot. *Bottom, querying*: queried elements are hashed, and the value of the corresponding slot is returned. Three situations may occur: **1/**true positive (e.g. “ACTGA”), **2/**false positive (e.g. “CTGAC”) or **3/**true negative (“TGACA”). A true positive is always either correct or overestimated (in case of a hash collision between two elements with different abundances).

There exist several models and implementations of counting AMQ. The simplest being the counting Bloom Filter (Fan et al. 2000) (cBF for short) using a unique hash function, of which a toy example is given in Fig. 1. In a cBF, each element is hashed to get a position in a bit vector, at which position its abundance is stored. This requires a few bits per entry for storing this abundance. Collisions are allowed: should a collision occur, the abundance stored is the maximum of the colliding elements. This leads to a non-null probability of overestimation. A cBF is a generalisation of Bloom filters, in which each element is allocated only one bit, recording its presence/absence. Hence, a cBF requires more memory than a Bloom filter to achieve the same false positive rate. Equivalently, a cBF has a higher false positive rate than a simple Bloom filter with constant memory.

It should be noted that other cAMQ exist, such as the counting quotient filters (Pandey et al. 2017). Despite the fact that the method we propose is applicable to any cAMQ, the description presented here, as well as the associated implemented tool, are based on the counting Bloom Filter using a unique hash function.

### 2.2 Overview of fimpera

#### 2.2.1 Core principle

fimpera’s objectives are to reduce FPR_cAMQ_ and to improve the precision of the reported abundance of true positive calls. This is achieved using a method based on splitting *k*-mers into smaller words called *s*-mers. The *s*-mers are the elements indexed in the counting AMQ at indexation time. At query time, a *k*-mer is reported as found if and only if every one of its *s*-mers is found in the counting AMQ. Thus, a false positive (resp. overestimation) on a *k*-mer requires k−s+1>1 false positives (resp. overestimations) on its *s*-mers.

Alternatively, for a fixed FPR_cAMQ_, the fimpera strategy reduces the memory needed by the cAMQ up to several orders of magnitudes.

Splitting *k*-mers into z+1 s-mers and associating each *s*-mer with a hash value as done in fimpera may be seen as a similar strategy as using H=z+1 hash functions per *k*-mer with a bloom filter. There are three obvious observations to be made here:

- Canonical indexing methods such as HowDeSBT (Harris and Medvedev 2020) or Cobs (Bingmann et al. 2019) for instance rely on bloom filters using a unique hash function. These state-of-the-art approaches can thus directly benefit from our proposal, with no modification.
- Indexing and query time grow linearly with the number of independent hash functions used, while fimpera takes advantage of the *s*-mers shared between consecutive *k*-mers. Even if it is not restricted to this framework, fimpera is meant for querying streams of *k*-mers. In this situation, when streaming all successive *k*-mers from a sequence, a *k*-mer shares z s-mers with its predecessor. Only one unique new *s*-mer has to be inserted or queried for all *k*-mers except the first one. Thus, with fimpera, the computation time does not grow with the *z* value. Notably, as shown in Table 2, it even slightly decreases at query time, as once a *s*-mer is detected as absent, all *k*-mers that span this *s*-mer can be skipped.
- Regarding the effect on memory, in a Bloom filter with *H* > 1 distinct hash functions, the *H* value is limited to an optimal as using too many hash functions saturates the filter load factor, leading either to an increase of the false positive rate or to the need of increasing the filter size. This is not the case when using the fimpera approach.

**Table 1.** Computation example of the *r* vector, with a window of size 3.^a^

*i*	0	1	2	3	4	5	6	7	8	9
*v*	5	3	7	1	4	5	3	2	2	3
*j*	0			1			2			3
${\text{min}}_{L}$	5	3	3	1	1	1	3	2	2	3
${\text{min}}_{R}$	3	3	7	1	4	5	2	2	2	3
r	3	**1**	1	1	3	2	2	2		

a Tables min_*L*_ and min_*R*_ are represented for helping comprehension but are not implicitly created in practice. The *j* row indicates the starting positions of each fixed windows. As an example, the minimal value of the sliding window of size 3 starting position *i *=* *1 is r[1]=1 (bold underlined value), being equal to $\min({\text{min}}_{L}[1+3-1],{\text{min}}_{R}[1])=\min(1,3)$ (underlined values).

**Table 2.** Influence of the z parameter on the quality of the results and on the computation time when indexing and querying 31-mers through their s-mers.^a^

z	0	3	5	7	9	20
(indexed s-mer size s=31−z)	31	28	26	24	22	11
False positive rate (%)	25.00	0.56	0.08	0.02	0.03	99.70
Of which: construction FP (%)	0	0.49	9.64	46.32	67.26	99.99
Incorrect abundance calls (%)	1.55	1.33	1.93	2.99	4.27	42.73
Of which: constr. overest. (%)	0	83.02	88.65	94.24	94.34	99.85
Query time (s)	557	514	498	491	493	528

a “*constr.*” stands for “*construction*” and “*overest.*” stands for “*overestimation*”. Results with z=0 are equivalent to those obtained with the original cBF results. The Incorrect abundance calls are computed over true positive calls only.

#### 2.2.2 Indexation overview

At indexation time, fimpera takes as input a file of counted *k*-mers, as provided for instance by KMC (Kokot et al. 2017). fimpera splits each *k*-mer into its k−s+1 constituent *s*-mers (k≥s). Each *s*-mer is then stored in a cAMQ along with its s-abundance, denoted as *s*_ab_. The *s*_ab_ of a *s*-mer is formally defined as the maximum of the abundance of the *k*-mers containing this *s*-mer. We explain the choice on relying on *s*_ab_ instead of the abundance of *s*-mers and describe its outcomes in the Supplementary Section S3.3. Using both the *s*_ab_ and the abundance of *s*-mers is supported in the implementation.

In the following, we set z=k−s, hence z≥0.

#### 2.2.3 Query overview

For each queried sequence *S*, fimpera extracts its sequence of *s*-mers, which is then queried against the cAMQ, and the abundance of any *k*-mer of *S* is computed as the minimum of *s*_ab_ of its *s*-mers. By default, fimpera prints each input sequence along with the abundance of every of its consecutive *k*-mer. In practice, the input query file is a fasta or a fastq file, possibly gzipped.

#### 2.2.4 False positive calls

Let’s consider a *k*-mer *d* with an abundance of 0 and each of its *s*-mer has an *s*_ab_ of 0 as well. With fimpera, wrongly reporting *d* as present requires that *every s*-mer of that *k*-mer are wrongly found as present in the counting AMQ. The probability of such an event is approximately $F{\left({\text{PR}}_{\text{cAMQ}}\right)}^{z+1}$, leading to a dramatic decrease in the occurrences of false positive calls with respect to *z*. For instance, with *z *=* *3 (which is a recommended and the default value) and a counting AMQ having a false positive rate of 25%, the false positive rate with fimpera for that setting is ≈0.4%.

The fimpera approach may generate a novel kind of false positive. A queried *k*-mer, absent from the indexed dataset, may be composed of *s*-mers existing in this indexed set. Querying such a *k*-mer with fimpera returns a non-zero abundance, so generating a false positive, which we call a “construction false positive”. These false positives are created by fimpera, *independently of the underlying cAMQ*. This event is non-null but it is in practice negligible when using usual *k* and *z* values, as shown in the results.

#### 2.2.5 Overestimations

To overestimate the abundance of a queried *k*-mer with fimpera, overestimations are required to happen on the abundance of **every** *s*-mer of that *k*-mer that has the minimal *s*_ab_. The more *s*-mer per *k*-mer, the more *s*-mer abundance overestimations need to happen to overestimate a *k*-mer abundance. *s*-mer abundance overestimations can come from two sources:

- a collision occurs in the counting Bloom Filter, leading to the overestimation of the less abundant colliding *s*-mer; and/or:
- a *s*-mer is shared among two different *k*-mers having different abundances. This overestimates the abundance of this *s*-mer from the least abundant *k*-mer. This happens no matter the false positive rate of the counting Bloom Filter. We call those overestimations “construction overestimation”. This new kind of overestimation is specific to fimpera.

Observe a case of interest: consider two *k*-mers *d*_0_ and *d*_1_ overlapping over *k –* 1 characters. If *d*_1_ has an abundance greater than *d*_0_, then the correct abundance of *d*_0_ is retrievable through a unique *s*-mer (the unique *s*-mer of *d*_0_ that does not appear in the *k*-mer *d*_1_). In such case, *d*_0_ is more likely to be overestimated than *d*_1_.

Consequently, fimpera’s overestimations are not uniformly distributed random events. Overestimations are more likely to occur close to a change in abundance along queried sequences than in a random *k*-mer. In such cases, those overestimations are limited to the abundance of their neighbor *k*-mers, mitigating their impact. Indeed, in the result section (Section 3), we show that the erroneous abundance calls are closer to the ground truth with fimpera compared to those obtained with the original cBF.

We now describe in more detail both the indexing step and the querying step of fimpera, as well as one optimisation, allowing a query time independent from the *z* value.

Another optimisation enables fimpera to perform queries slightly faster than using the original underlying cAMQ. This optimisation is described in Supplementary Section S2.2.

### 2.3 Querying with fimpera

fimpera’s query consists in querying all consecutive, overlapping *k*-mers from a sequence of size greater than or equal to *k* through their constituent *s*-mers. fimpera’s query is a two-step process:

- for every position in the query except the last *s—*1 ones, *s*-mers starting at these positions are queried in the counting AMQ and are stored in an array of integers *s*_ab_;
- the abundance of any *k*-mer starting position *p* is the minimum value of the sub-array of length (*z* + 1) starting at the position *p*: $s_{\text{ab}}[p;p+z]$.

A non-optimised version of the fimpera’s query algorithm is shown in Supplementary Section S1.

The first step is improved by avoiding recomputing the minimal value of windows of length *z *+* *1 starting at each position *p* of an array. This optimisation is described in the following section.

### 2.4 Optimisation: sliding window minimum algorithm

The problem, independent of fimpera, is as follows: given a vector of values (integers or floats) *v* and an integer *W* (denoting the size of a sliding window, with W≤|v|), give an array *r* such that ∀i∈[0,|v|−W],r[i]=min(v[i],v[i+1],…,v[i+W−1]). This is a particular case of a more general problem, the “range minimum query” (RMQ). Given a vector *v* of element drawn from a totally ordered set and two integers *i*, *j* (0≤i<j<|v|), the RMQ consists in finding min(v[i],v[i+1],…,v[j−1]. Answering RMQ generally relies on some pre-computation beforehand [e.g. pre-computing the whole set of possible queries, but less resource-intensive solutions can be found, such as Durocher and Singh (2019)].

The sliding window minimum problem studied here is a particular case of an RMQ (*j – i* is fixed and queries consist in every window of that size). Thus, its solutions do not require taking into account other window sizes, effectively allowing to skip pre-computation. The naive approach to solving the sliding minimum problem is to simply search for the minimal value in each window. This algorithm is in O(W×|v|) time. Some straightforward faster solutions can be built on top of dynamic heaps. Here, we propose a solution (named “fixed window”) that does not rely on any heap allocation (which is slow for most systems). This solution without any dynamic memory allocation is an order of magnitude faster than other O(|v|) solutions as shown Supplementary Section S3.1 in the Supplementary Materials.

The main idea of the proposed “fixed window” approach is to split the input vector of values in fixed, non-overlapping windows of size *W*. Then, for each so-called “fixed window”, compute two vectors:

-
${\text{min}}_{L}\_j$ : ${\text{min}}_{L}\_j[i\%W]$ contains the minimum value encountered in the *j*th fixed window up to the position *i*;(i∈[i×W×j;i×W×(j+1)−1]
-
${\text{min}}_{R}\_j$ : ${\text{min}}_{R}\_j[i\%W]$ contains the minimum value from the position *i* up to the end of the *j*th fixed window.

All ${\text{min}}_{L}\_j$ and ${\text{min}}_{R}\_j$ vectors are then concatenated into two vectors (min_*L*_ and min_*R*_). The minimum of a *sliding* window starting at position *i*, denoted by r[i], is thereupon the minimum between:

-
${\text{min}}_{L}[i+W-1]$ (the minimum of the left part of the next fixed window)
-
${\text{min}}_{R}[i]$ (the minimum of the right part of the current fixed window)

An example is provided in Table 1.

Note that, as described previously, this approach would require allocating memory for two vectors per call. This memory need may appear negligible in theory as those vectors are limited by the query size which is a few hundred to few thousand. However, in practice, allocating memory for these vectors is time-consuming, and may increase significantly the practical running time. We overcame this memory need thanks to these three following tricks. **1/**we compute ${\text{min}}_{L}[i]$ on the fly (if i%W≠0 then ${\text{min}}_{L}[i]=\text{min}({\text{min}}_{L}[i-1],v[i])$, else ${\text{min}}_{L}[i]=v[i]$). **2/**min_*R*_ is computed *directly in the queried vector*. This does not impact the correctness of the algorithm, as $r[i]\leq {\text{min}}_{R}[i]\leq v[i]$. **3/**the response (minimal value per sliding window) can be stored directly in the input queried vector as well. At the price of modifying the input vector, this allows the algorithm to be run in O(size_query) time while avoiding any time-consuming heap allocation.

A complete description of the optimised solution is provided in Supplementary Section S2.1, Supplementary Algorithm S2, along with a benchmark Supplementary Section S3.1, Supplementary Fig. S1.

This algorithm offers a generic solution for computing the minimal value of a sliding window in constant memory and linear time. Its usefulness is not limited to fimpera. As so, we propose an independent implementation https://github.com/lrobidou/sliding-minimum-windows. Note also that it can be straightforwardly modified for computing the maximal value instead of the minimal value of each window.

### 2.5 Implementation of fimpera

An implementation of fimpera is available at https://github.com/lrobidou/fimpera. This implementation is specialised for genomic data (i.e. with an alphabet consisting of A, T, C, G) and uses a counting Bloom Filter with a unique hash function as cAMQ. A template mechanism allows the use of any other cAMQ provided by the user. Queries consist of fasta or fastq files (gzipped or not), and an option is provided to index and query canonical *k*-mers only, i.e. the lexicographic minimum between each *k*-mer and its reverse complements. Indexing options include the *k* and *z* values, the size of the filter, and *b*, the number of bits per element used to store its abundance. As *b* has a major impact on the final size of the data structure, it is recommended to use low *b* values (say b≤5). This limits the maximal stored abundance value to $2^{b}-1$ (to prevent overflow, the abundance is capped at $2^{b}-1$).

In order to encode large abundance values with few bits per *k*-mer, instead of storing the first possible $2^{b}-1$ distinct abundance values, we propose to discretize any abundance value to user-defined interval ranges. In practice, fimpera can use any surjective function


$$f(x\in [1,\infty ])\to y\in [0,2^{b}-1],$$

supplied by the user. This can be for instance any intervals. The proposed implementation proposes the usage of $y=\min(\left\lfloor {\;\text{log}\;}_{2}(x)\right\rfloor ,2^{b}-1)$, or $y=\min(\left\lfloor {\;\text{log}\;}_{10}(x)\right\rfloor ,2^{b}-1)$.

Changing the default output (e.g. storing results instead of printing them, computing average abundance per sequence, or printing only sequences whose average *k*-mer abundances is above a user-defined threshold) is possible.

## 3 Results

### 3.1 Experimental setup

To the best of our knowledge, no other tool focuses on reducing the false positive rate of existing cAMQ, thus we compare fimpera results applied on a counting Bloom Filter indexing *s*-mers with the original counting Bloom Filter results indexing *k*-mers using a unique hash function. We propose results on biological marine metagenomic data.

A list of commands for reproducing the results is available here: https://github.com/lrobidou/fimpera/blob/paper/paper\_companion/Readme.md along with a step-by-step explanation of the output. Executions were performed on the GenOuest platform on a node with 4x8cores Xeon E5-2660 2.20 GHz with 200 GB of memory.

### 3.2 Metagenomic dataset

We used two fastq files from the TARA ocean metagenomic dataset (Sunagawa et al. 2020) to show the advantages offered by fimpera on metagenomic samples. The index was computed from the $2.38\times 10^{8}$ distinct 31-mers present at least twice in an arctic station (accession number ERR1726642) and the query sample was the first $3\times 10^{6}$ reads from a sample in another arctic station (accession number ERR4691696). Canonical *k*-mers were considered for this experiment.

### 3.3 Choice of filters parameters

In this experiment, we apply the fimpera approach on top of a counting Bloom Filter designed to have 25% of false positive calls, while using 5 bits per element for storing the abundance of indexed *k*-mers. For indexing $2.38\times 10^{8}\;31$-mers this structure requires $6.96\times 10^{8}$ slots (thus $3.48\times 10^{9}$ bits).

We compare the results of queries made against this raw counting Bloom Filter with the results of queries made using fimpera wrapping that same counting Bloom Filter.

Thus, parameters used are as follow: *k *=* *31, size of the filter of $3.48\times 10^{9}$ bits, as discussed in Section 3.3, using *b *=* *5 bits per abundance count (thus $2\times 10^{8}$ slots), and abundances are stored as their $\left\lfloor {\;\text{log}\;}_{2}\right\rfloor$ values. We use the default *z *=* *3 parameter (unless otherwise stated). As we use *z *=* *3, we compare the results of a cBF indexing 31-mers, with results of fimpera used on a cBF with the same sizing, but indexing *s*-mers of size 28 (31-3).

### 3.4 Used metrics

To measure the quality of the fimpera results and the cBF results, we propose three metrics:

- the false positive rate, which provides the probability that the method returns an abundance call > 0 for a *k*-mer absent from the indexed set.
- the proportion of incorrect abundance, that provides the probability that the method returns the incorrect abundance for a *k*-mer actually in the indexed set.
- statistics of responses for incorrect abundances calls, that estimate the reported abundance of *k*-mers whose abundance is incorrectly reported. When comparing any two approaches, we set up a metric that we call “overestimation score”. It is defined as the sum of the square of errors made on the output of each method. The lower the overestimation score is, the fewer errors were made. Errors that are closer to the ground truth are less penalised by this score than errors distant from it.

### 3.5 False positive rate analyses

**Figure 2.**
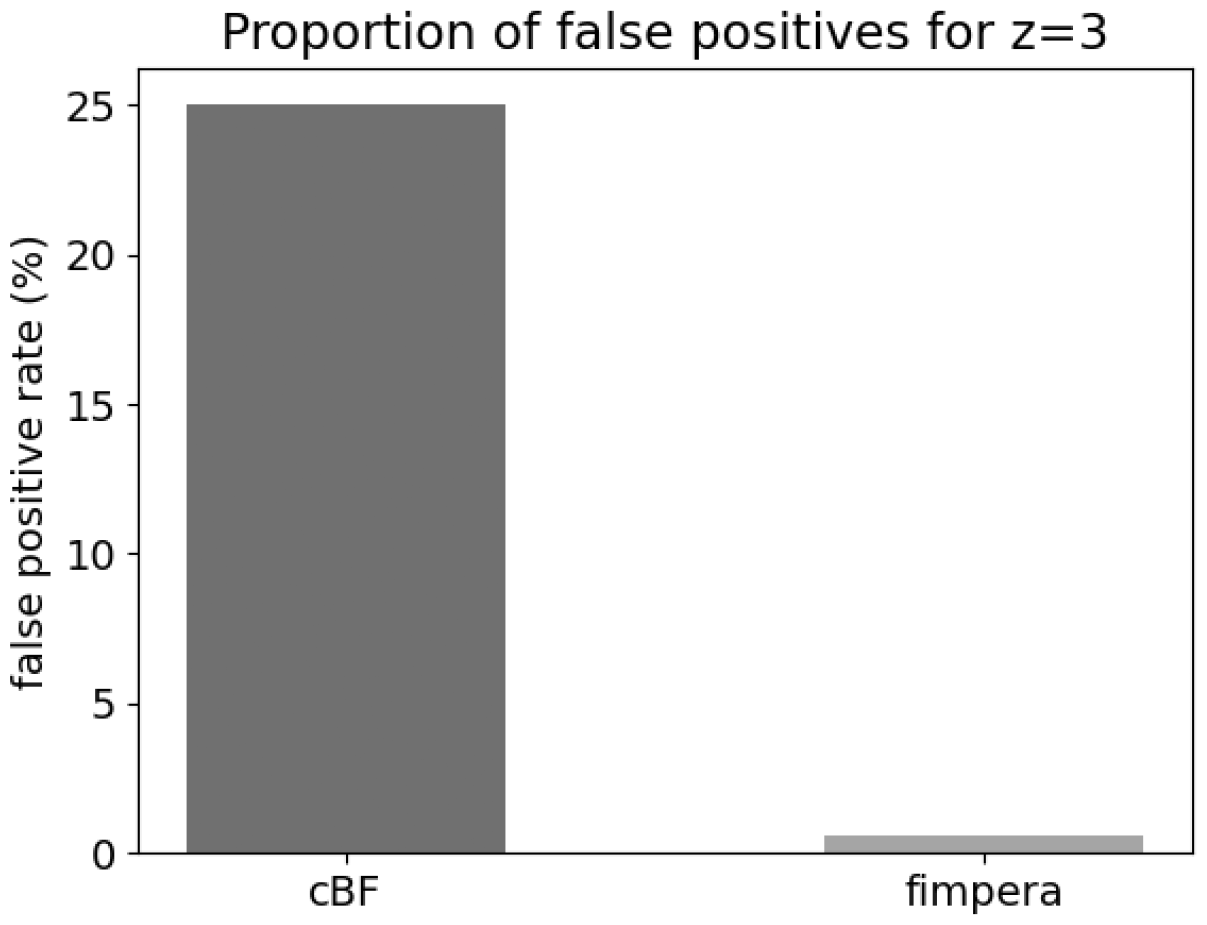
Proportion of false positive calls without fimpera (on a classical counting Bloom Filter) and with fimpera (*z *=* *3), indexing and querying real metagenomic read datasets.

Results about false positives obtained with the proposed experiment are shown in Fig. 2. Results about the cBF simply confirm the setup and show a false positive rate of 25%. When applying fimpera, the false positive rate drops to 0.56%. Among all these fimpera false positives, 4.8% are due to the so-called “construction false positives” (see Section 2.2.3), thus representing 0.0027% of the total *k*-mer calls.

It is important to recall that these comparative results were obtained using the exact same amount of space. Hence the fimpera approach enabled to yield about 45 times fewer false positive calls, with no drawback and even saving query time (see Table 2).

### 3.6 Correctness of the reported abundances

In this section, we focus only on true positive calls. Hence, these results do not concern the 25% false positive calls obtained with the original cBF, nor the 0.56% ones using fimpera.

Results comparing the proportion of calls reported with an incorrect abundance among the true positives show that 1.54% of true-positive calls are overestimated in the cBF, while 1.33% of true-positive calls are overestimated with fimpera. Among the fimpera calls estimating an incorrect abundance among the true positives, 83% are due to the so-called “construction overestimation”.

### 3.7 Distribution of errors in overestimated calls

In this section, we focus only on the wrongly estimated calls among true positives.

**Figure 3.**
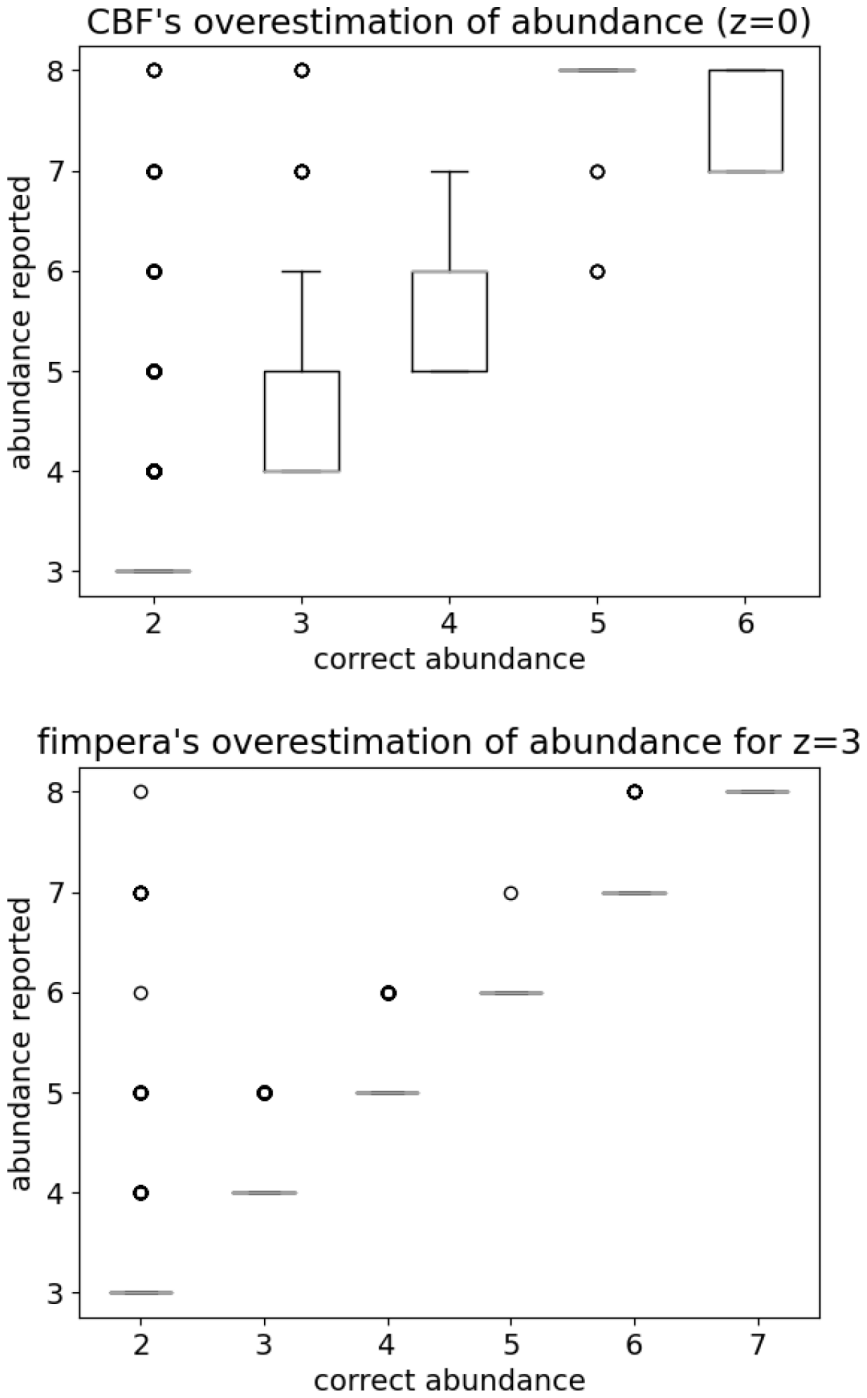
For true-positive calls with an incorrect abundance estimation: reported abundance with respect to the correct abundance. Top: using the original cBF, Bottom: using fimpera.

Results presented Fig. 3 show that, as stated in Section 2.2.5, the erroneous abundance calls are closer to the ground truth with fimpera compared to those obtained with the original cBF. As seen Fig. 3-bottom, with fimpera, almost all (except for a few outliers) overestimations are only one value apart from the correct range (the average difference with the correct abundance range is 1.07). With the original cBF, as seen Fig. 3-left, overestimations are more important (1.33 range in average from the ground truth).

### 3.8 Influence of the size of the underlying cAMQ

Because there is a trade-off between space usage and the false positive rate of a cAMQ, fimpera can either reduce the false positive rate of the cAMQ without changing its size or reduce its size without changing its false positive rate. This section focuses on the impact of fimpera on this latter strategy.

**Figure 4.**
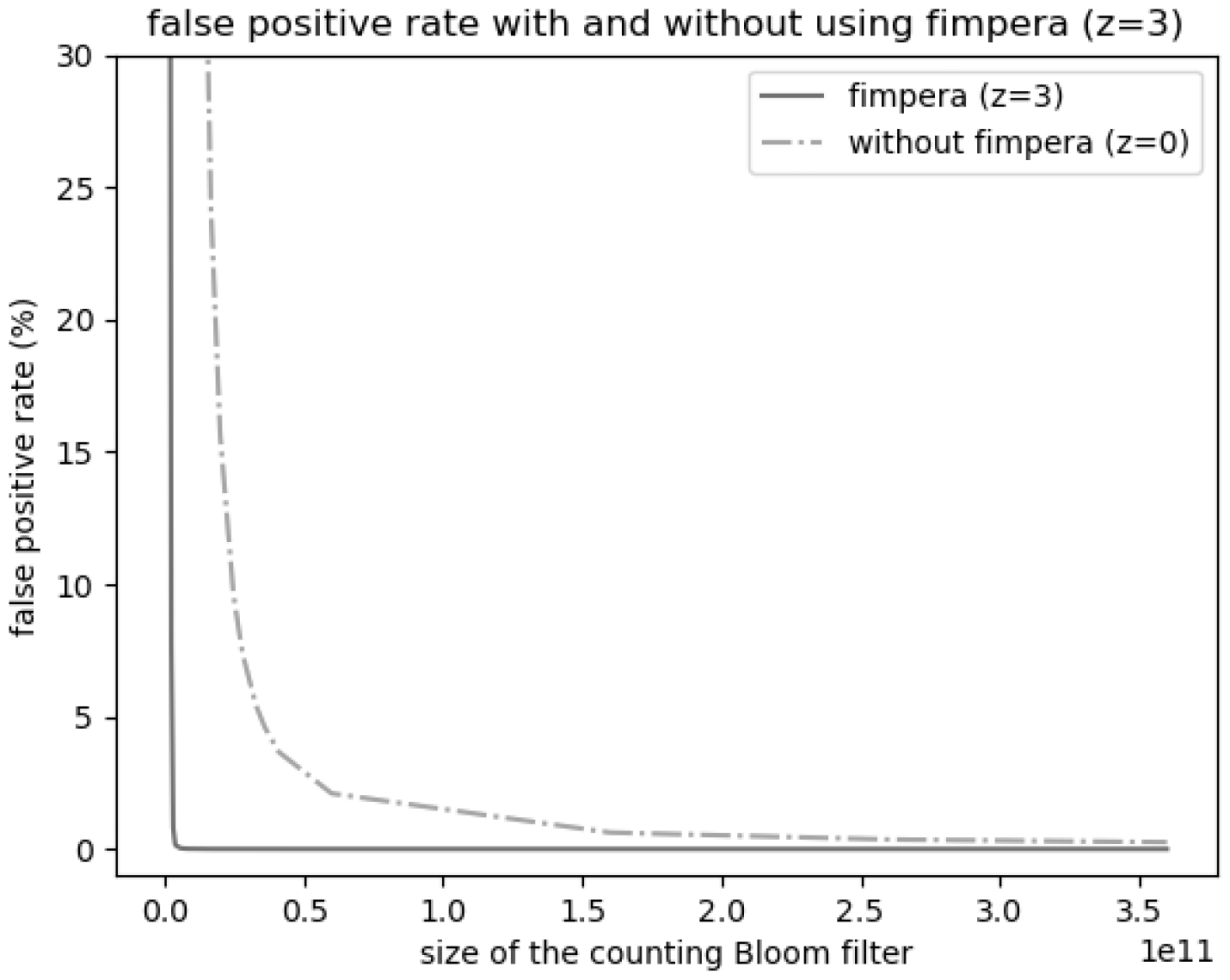
Variation of the false positive rate with respect to the size of the cBF, with and without fimpera (*z *=* *3). As we consider here the average abundance of *k*-mers on each queried read, a false positive means that the average abundance of a *k*-mer on a read is strictly positive while it should have been 0.

**Figure 5.**
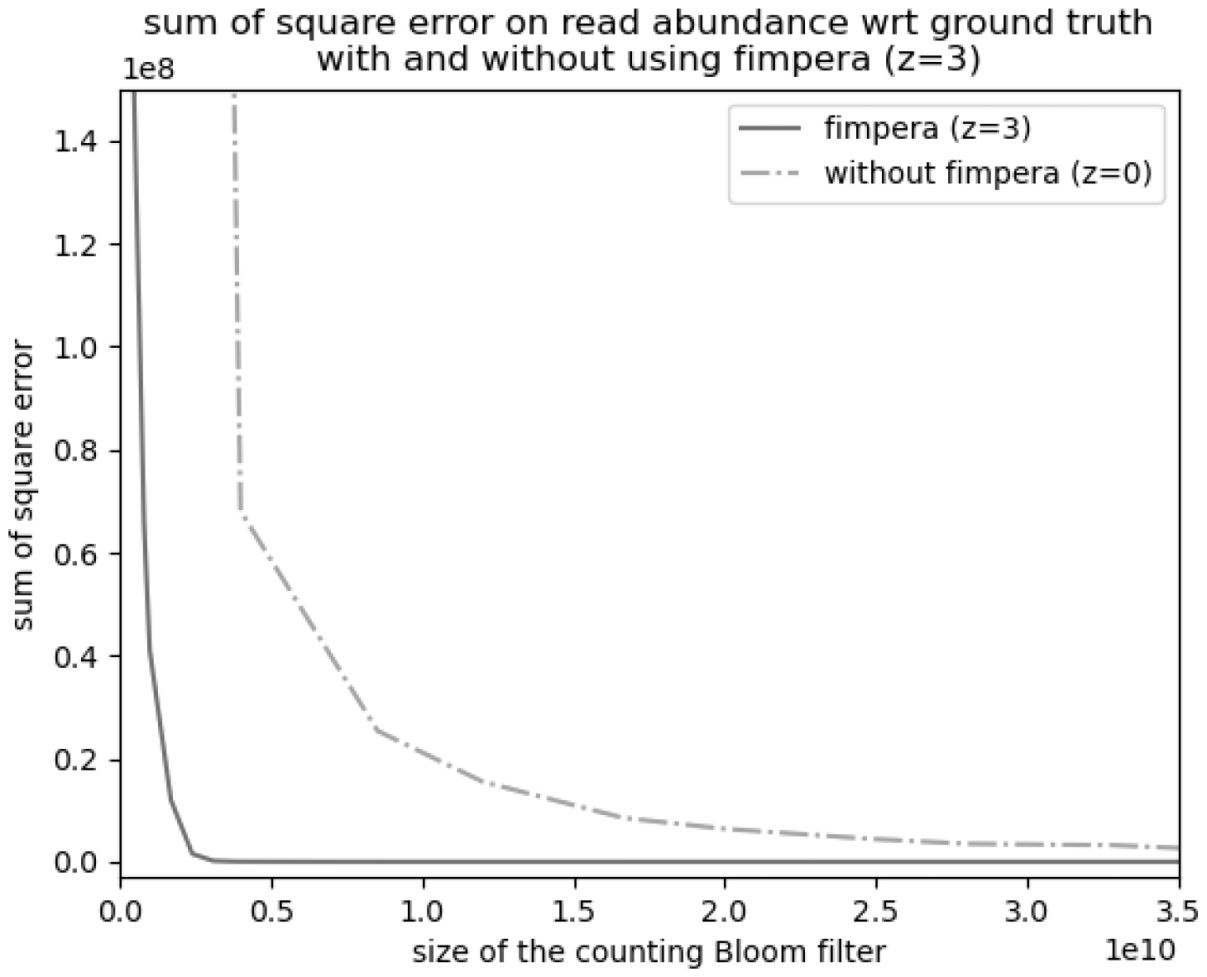
Sum of square error with respect to the size of the cBF, with and without fimpera (*z *=* *3). In this experiment, the output of fimpera is the *average of each input read*.

This trade-off (with and without fimpera) is shown Figs 4 and 5. To be as close as possible to real-life use-case, we consider in this section the average abundance of *k*-mer on each read, not the abundance of each *k*-mer. For precision requirement, abundances were not stored as their $\left\lfloor {\;\text{log}\;}_{2}\right\rfloor$ values, but rather as their original values.

With fimpera, achieving a false positive rate of 2% requires $\approx 2.98\times 10^{9}$ bits ($\approx 3.72\times 10^{8}$ slots of 8 bits each) (see Fig. 4). Without fimpera, achieving the same false positive rate requires $\approx 65.5\times 10^{9}$ bits (21 times more space). The lower the false positive rate, the greater the space gap between using and not using fimpera. Achieving a false positive rate of 0.02% requires about $7.25\times 10^{9}$ bits with fimpera, but $360\times 10^{9}$ bits were not enough without fimpera (i.e. allocating more than 50 times the space budget of fimpera would be required). In order to take into account the abundance information, we set up the output of fimpera to be the average of *k*-mers’ abundance for each queried read.

Figure 5 shows the sum of square of overestimations, with and without fimpera. For an overestimation score (defined Section 3.4) of 200000, fimpera requires $\approx 3.10\times 10^{9}$ bits ($\approx 3.87\times 10^{8}$ slots of 8 bits each), whereas a cBF would requires $330.9\times 10^{9}$ bits (i.e. 100 times more space).

#### 3.8.1 Impact of *z*

As shown in Table 2, the false positive rate decreases with respect to *z* and stays low for a wide range of *z* values (at least from *z *=* *3 to *z *=* *9). When using an extreme *z* value, for instance, *z *=* *20, the false positive rate is increased up to almost 100%. With *z *=* *20, as we use *k *=* *31, the size of the indexed *s*-mers is *s *=* *11. When indexing as little as a few hundred million characters, each 11-mer has a great probability to appear by chance in the indexed dataset. Indeed, the probability of such event is $1-{\left(1-\frac{1}{|\Sigma {|}^{s}}\right)}^{n}$, with *n* being the number of characters. For instance, for *s *=* *11 and a sequence as little as n=20 million characters, the probability of the presence of any 11-mer is > 99%. This quasi-random existence of all *s*-mers generates a huge amount of construction false positives, as seen in the last column. This has an effect on the running time, which is close to the query time of *z *=* *0, undoubtedly because all *s*-mers queried are positives, annihilating the *s*-mer skipping optimisation.

Caveat: in Table 2, the overestimations are reported only for true positive calls. Overall, also taking into account the negative answers, the overestimation rate with fimpera would be 0.03 with *z *=* *3 for instance.

In the Supplementary Section S3.4, we show the impact of *z* in a different setup: choosing a fixed *s* and increasing *k* with regard to *z*, instead of fixing *k* and decreasing *s* with regard to *z*. This shows another framework for using fimpera, with similar conclusions about the quality of the results.

#### 3.8.2 Query time

As mentioned in the Supplementary Section S2.2, the fimpera approach does not increase the query execution time. On the contrary, it makes it possible to reduce the running time slightly as *z* increases. See Table 2.

#### 3.8.3 Discussion

A Bloom filter indexing $2.38\times 10^{8}$ elements using $2.33\times 10^{8}$ bits and one hash function would lead to a false positive rate of ≈6.61%. A counting Bloom Filter indexing the same dataset using the same space with 5 bits per slot (thus 5 times fewer slots) yields a false positive rate of 25%. With fimpera, using 5 bits per slot and the same total number of bits, the false positive rate drops to 0.56%. Using the same space as a Bloom filter, fimpera allows reducing the false positive rate, while adding count storage and being quicker to query. The only downside may in some cases be the computation of the *s_ab_* from the abundance of counted *k*-mers. However, this can be skipped altogether with a moderate impact on the result (see Supplementary Section S3.3).

## 4 Conclusion

We presented fimpera, a novel computational method for reducing the false positive rate and increasing precision in any counting AMQ data structure. This is achieved without requiring any changes to the original data structure, with no memory overhead, and even with a slight improvement in query computation time.

Our results showed that when applied on top of a counting Bloom Filter, fimpera enabled to yield about 45 times fewer false positive calls than when querying directly a counting Bloom Filter of identical size. Moreover, using fimpera, abundance errors were slightly less frequent on true positive calls, and finally, those abundance errors were on average 1.07 apart from the ground truth with fimpera while they are on average 1.33 apart from the ground truth with the original cBF.

Independently from parameters of the used cAMQ, fimpera requires setting up a unique parameter, *z*. Fortunately, results are highly robust with the choice of *z*, unless extreme values are chosen. Future work will include a formal analysis of the theoretical limits on the choice of *z* usage ranges.

We provide a C++ implementation of fimpera which enabled us to validate the approach. This implementation can also be used as a stand-alone tool for indexing and querying genomic datasets, and it can be tuned with user-defined parameters and ranges of abundances. The provided GitHub project also proposes all necessary instructions and links to genomic data to reproduce the results.

Finally, of independent interest, we proposed a novel algorithm and its implementation for computing the minimal or maximal values of consecutive windows, sliding on an array of integers or floats. To the best of our knowledge, this is the fastest algorithm to perform this task.

## Supplementary Material

btad305_Supplementary_Data

## References

[btad305-B1] Alanko JN , Simon JP, Jaakko V. Succinct k-mer sets using subset rank queries on the spectral burrows-wheeler transform. *bioRxiv* 2022;2022–05.

[btad305-B2] Altschul SF , Gish W, Miller W et al Basic local alignment search tool. J Mol Biol 1990;215:403–10. 2231712 10.1016/S0022-2836(05)80360-2

[btad305-B3] Bingmann T , Bradley P, Gauger F, Iqbal Z. COBS: a compact bit-sliced signature index. In: *International Symposium on String Processing and Information Retrieval*, SPIRE 2019, pp. 285–303. Springer.

[btad305-B4] Bowe A , Onodera T, Sadakane K, Shibuya T. Succinct de bruijn graphs. In: *Algorithms in Bioinformatics: 12th International Workshop, WABI 2012*, September 10–12, 2012, Ljubljana, Slovenia: Springer, 2012, pp. 225–235.

[btad305-B5] Bradley P , den Bakker HC, Rocha EPC et al Ultrafast search of all deposited bacterial and viral genomic data. Nat Biotechnol 2019;37:152–9. 30718882 10.1038/s41587-018-0010-1 PMC6420049

[btad305-B6] Chikhi R , Holub J, Medvedev P et al Data structures to represent a set of k-long DNA sequences. ACM Comput Surv 2022;54:1–22. 10.1145/3445967 PMC12965495 41798316

[btad305-B7] Cummins C , Ahamed A, Aslam R et al The European nucleotide archive in 2021. Nucleic Acids Res 2022;50:D106–D110. 34850158 10.1093/nar/gkab1051 PMC8728206

[btad305-B8] Durocher S , Singh R. A simple linear-space data structure for constant-time range minimum query. Theor Comput Sci 2019;770:51–61.

[btad305-B9] Fan L , Cao P, Almeida J, Broder AZ. Summary cache: a scalable wide-area web cache sharing protocol. IEEE/ACM Trans Network 2000;8:281–93.

[btad305-B10] Harris RS , Medvedev P. Improved representation of sequence bloom trees. Bioinformatics 2020;36:721–7. 31504157 10.1093/bioinformatics/btz662 PMC8215923

[btad305-B11] Kokot M , Dlugosz M, Deorowicz S et al KMC 3: counting and manipulating k-mer statistics. Bioinformatics 2017;33:2759–61. 28472236 10.1093/bioinformatics/btx304

[btad305-B12] Limasset A , Rizk G, Chikhi R, Peterlongo P. Fast and scalable minimal perfect hashing for massive key sets. In: *16th International Symposium on Experimental Algorithms (SEA 2017), volume 75 of Leibniz International Proceedings in Informatics (LIPIcs)*, pp. 25:1–25:16. Dagstuhl, Germany: Schloss Dagstuhl–Leibniz–Zentrum fuer Informatik, 2017.

[btad305-B13] Marchet C, Kerbiriou M, Limasset A. Efficient exact associative structure for sequencing data. In: Recomb-Seq 2019-9th RECOMB Satellite Workshop on Massively Parallel Sequencing, 2019 May 3 (pp. 1–16).

[btad305-B14] Marchet C , Boucher C, Puglisi SJ et al Data structures based on k-mers for querying large collections of sequencing data sets. Genome Res 2021;31:1–12. 33328168 10.1101/gr.260604.119 PMC7849385

[btad305-B15] Pandey P , Bender MA, Johnson R, Patro R. A general-purpose counting filter: Making every bit count. In: *Proceedings of the 2017 ACM International Conference on Management of Data*, pp. 775–787, 2017.

[btad305-B16] Pibiri GE. On Weighted k-mer Dictionaries. In: Boucher C, Rahmann S (eds.), *22nd International Workshop on Algorithms in Bioinformatics (WABI 2022)*, volume 242 of Leibniz International Proceedings in Informatics (LIPIcs), pp. 9:1–9:20, Dagstuhl, Germany: Schloss Dagstuhl – Leibniz-Zentrum für Informatik, 2022a.

[btad305-B17] Pibiri GE. Sparse and skew hashing of k-mers. Bioinformatics2022b;38:i185–i194. 35758794 10.1093/bioinformatics/btac245 PMC9235479

[btad305-B18] Pibiri GE , Trani R. 2021. PTHash: Revisiting FCH minimal perfect hashing. In: *Proceedings of the 44th International ACM SIGIR Conference on Research and Development in Information Retrieval*, pp. 1339–1348.

[btad305-B19] Rahman A , Medvedev P. Representation of k-mer sets using spectrum-preserving string sets. Technical report. Cold Spring Harbor Laboratory. New Results, 2020.

[btad305-B20] Robidou L , Peterlongo P. findere: fast and precise approximate membership query. In: *International Symposium on String Processing and Information Retrieval*, pp. 151–163. Springer, 2021.

[btad305-B21] Shibuya Y , Belazzougui D, Kucherov G et al Set-min sketch: a probabilistic map for power-law distributions with application to k-mer annotation. J Comput Biol2022a;29:140–54. 35049334 10.1089/cmb.2021.0429

[btad305-B22] Shibuya Y , Belazzougui D, Kucherov G et al Space-efficient representation of genomic k-mer count tables. Algorithms Mol Biol2022b;17:5. 35317833 10.1186/s13015-022-00212-0 PMC8939220

[btad305-B23] Solomon B , Kingsford C. Fast search of thousands of short-read sequencing experiments. Nat Biotechnol 2016;34:300–2. 26854477 10.1038/nbt.3442 PMC4804353

[btad305-B24] Sunagawa S , Acinas SG, Bork P et al; Tara Oceans Coordinators. Tara oceans: towards global ocean ecosystems biology. Nat Rev Microbiol 2020;18:428–45. 32398798 10.1038/s41579-020-0364-5

[btad305-B25] Tomczak K , Czerwińska P, Wiznerowicz M et al The cancer genome atlas (TCGA): an immeasurable source of knowledge. Contemp Oncol 2015;1A:68–77. 10.5114/wo.2014.47136 PMC4322527 25691825

