# Supplementary material for: fimpera: drastic improvement of Approximate Membership Query data-structures with counts
Source: Bioinformatics. 2023 May 17;39(5):btad305. doi: 10.1093/bioinformatics/btad305 (PMC10212535; doi:10.1093/bioinformatics/btad305)
Supplement: btad305_Supplementary_Data [file btad305_supplementary_data.pdf]

Supplementary Materials
`fimpera`: drastic improvement of Approximate Membership Query data-structures with counts

Lucas Robidou and Pierre Peterlongo

These supplementary materials first propose a detailed description of the proposed query algorithms. We start by providing the simple naive query algorithm (Section 1). Then, we present two optimization approaches (Section 2.1 and 2.2). These supplementary materials also propose additional results:

- showing the advantages of the sliding window algorithm we propose 3.1,
- showing a comparison between using `fimpera` on a counting AMQ having a unique hash function, or not using `fimpera` on a counting AMQ with $z+1$ hash functions (Section 3.2),
- showing the effects of using the abundance of s-mers, instead of their s-abundances (Section 3.3),
- showing the effects of choosing the k value at query time and not at indexing time (Section 3.4),
- and showing the effects of not grouping values using log functions (Section 3.5).

1 Query algorithm: non-optimized version

A non-optimized algorithm of `fimpera`'s query is shown in Algorithm 1. This algorithm takes a queried sequence q, a counting AMQ indexing s-mers, and parameters k and z. It returns a vector of integers called *response*, such that: $\forall i \in [0, |response|-1]$, $response[i]$ is the abundance of the k-mer starting at position i in the query, for all i in $[0, |q|-k+1]$.

Algorithm 1 Non optimized `fimpera`'s query

1: **procedure** NON_OPT_QUERY($q \in \Sigma^*$; cAMQ indexing s-mers; k and z in $\mathbb{N}^+$) with $|q| \geq k, z < k$.)
2: ▷ Store the s-abundance of all s-mers from q:
3: $s_{abs} \leftarrow emptyVector(|q|-s+1)$
4: **for** i in $[0; |q|-s+1]$ **do**
5: $s_{abs}[i] \leftarrow get\_s_{ab}(q[i, i+s-1])$
6: ▷ get_s_{ab} uses the cAMQ to provide the s-abundances of a s-mer
7: **end for**
8: ▷ Compute all k-mers abundances from s-mers s-abundances:
9: $response \leftarrow emptyVector(|q|-k+1)$
10: **for** i in $[0; |q|-k+1]$ **do**
11: $response[i] \leftarrow \min_{j\in[i,i+z]}(s_{abs}[j])$
12: **end for**
13: **return** *response*
14: **end procedure**

Algorithm 1 is not optimal. Line 11 computes the minimal value of a range of z consecutive integers taken from a vector of integers. At each iteration of the *for loop* line 10, this range is shifted by one.

2 Optimized query algorithm

We propose two optimizations of the previous algorithm.

- The first one consists in using a minimum sliding window, that provides a way to compute in $O(1)$ the *min* from Line 11 of the non-optimized version of the query algorithm. This optimization is explained at a high level in the main text (Section 2.4). Here, Section 2.1 we provide the associated pseudo-code.
- The second optimization consists in exploiting the fact that for any queried s-mer detected as absent, all k-mers that contain this s-mer are also predicted as absent. Section 2.2 shows how to optimize the queries jumping all k-mer positions that contain an absent s-mer. This section finally proposes the overall optimized query algorithm 3.

2.1 Sliding window minimums

Algorithm 2 details our proposal for computing all minimal values of a sliding window of a vector of integers v in linear time and with zero memory allocation. Note that modifying this algorithm for computing the maximal values instead of minimal values is straightforward. Section 3.1, we propose results showing the advantages of this proposed algorithm.

Algorithm 2 sliding_minimum_window

```
procedure SLIDING_MINIMUM_WINDOW(vector v of positive integers; length of window w (|v| ≥ w, w > 1))
    nbWin ← ⌊size(v)/w⌋
    nb_elem_last_window ← |v| mod w
        ▷ Computation of min_left (See Section 2.4 in main text)
    min_left ← v[0]
    for i in [0; w − 1] do
        min_left ← min(min_left, v[i])
    end for
    for i in [0; nbWin − 2] do                          ▷ for every window excluding the last one
        start_window ← i × w
        for index in [start_window + w − 2; start_window] do          ▷ decreasing order
            v[index] ← min(v[index + 1], v[index])          ▷ compute min_right, directly in v
        end for
        for j in [0; w-1] do
                ▷ sliding_minimum is min(min_left, min_right(=v)):
            v[start_window + j] ← min(v[start_window + j], min_left)
                ▷ update min_left (if a new fixed window starts, reset min_left):
            if j == 0 then
                min_left ← v[start_window + w]
            else
                min_left ← min(min_left, v[start_window + w + j])
            end if
        end for
    end for
        ▷ Computation for the last window is not described here for the sake of simplicity
        ▷ remove last w − 1 elements from v:
    for i in [0; w − 2] do
        v.pop_back()
    end for
    return v
end procedure
```

2.2 Skip stretches of consecutive absent k-mers

Knowing the absence of a s-mer allows deducing that all k-mers containing this s-mer are absent. This allows inferring the existence of a stretch of consecutive absent k-mers.

We exploit this simple idea further. If one detects that two absent s-mers are $z+1$ positions away in the query, then any k-mer starting at any position between them is also absent. In the `fimpera` algorithm, if a s-mer is not found during the query, an optimization consists of searching for the abundance of the s-mer $z+1$ positions further away in the query. If that s-mer is also absent, there is no need to query any s-mer in between.

Thus, `fimpera` only needs to query one s-mer every $z+1$ position as long as the queried s-mers are absent in the counting AMQ, effectively saving time.

We can now propose the entire algorithm of `fimpera` (algorithm 3), using the sliding minimal window algorithm and including the optimization of skipping stretches of absent k-mers. The skip optimization occurs line 31: if a negative s-mer is called, the algorithm jumps $z+1$ position away in the sequence, probing for another absent s-mer. A positive answer triggers line 17, backtracking z positions backward. In this situation, we keep track of the fact that we are skipping s-mers via the *extending_stretch* flag.

Algorithm 3 Optimized `fimpera`'s query
($q \in \Sigma^*$; cAMQ indexing s-mers; k and z in $\mathbb{N}^+$) with $|q| \geq k, z < k$.

```
1:  s ← k − z
2:  response ← emptyVector(size − K + 1)
3:  stretchLength ← 0
4:  j ← 0                                              ▷ Current position in the query
5:  extending_stretch ← true
6:  previous_answers ← emptyVector(0)
7:  while j < |q| − k + 1 do
8:      smer ← q[j, j + s − 1]
9:      amq_answer ← s_ab_of_smer_in_cAMQ
10:     if amq_answer > 0 then
11:         if extending_stretch then
12:             previous_answers.push_back(amq_answer)
13:             stretchLength ← stretchLength + 1
14:             j ← j + 1
15:         else
16:             extending_stretch ← True
17:             j ← j − z
18:         end if
19:     else
20:         if stretchLength > z then
21:             start_of_stretch ← j − stretchLength
22:             offset ← 0
23:             for minimum : sliding_window_minimum(previous_answers, z + 1) do
24:                 response[start_of_stretch + offset] ← minimum
25:                 offset ← offset + 1
26:             end for
27:         end if
28:         previous_answers ← emptyVector(0)
29:         stretchLength ← 0
30:         extending_stretch ← false
31:         j ← j + z + 1
32:     end if
33: end while
34: if stretchLength > z then
35:     start_of_stretch ← |q| − k + 1 − stretchLength;
36:     offset ← 0
37:     for minimum : sliding_window_minimum(previous_answers, z + 1) do
```

```
        response[start_of_stretch + offset] ← minimum
        offset ← offset + 1
    end for
end if
return response
```

3 Additional results

In this section we propose additional results, highlighting the advantages of the new algorithm we propose for computing the minimal value of each sliding window (Section 3.1), or studying the connection between using `fimpera` or using a simple bloom filter with $z+1$ hash functions (Section 3.2). Finally, we propose additional results focused on the effect of using the s_{ab} instead of the abundance of s-mers (Section 3.3).

3.1 Sliding window minimum benchmark

We propose a benchmark of the following algorithms to compare algorithms that find the minimal value of each sliding window on a vector of integers v:

- "*recomputing*": a naive algorithm, computing naively the minimum in each window. Time: $\mathcal{O}(size\_window \times |v|)$.
- "*recomputing from last min*": keeping a reference to the minimum m in the previous window and recomputing the minimum in the current window only if the element of value m was the first element of the previous sliding window. Simply update m if the new element in the current window is smaller than m. Time: $\mathcal{O}(size\_window \times |v|)$ at worst (if v is an increasing sequence).
- a deque-based approach: the deque contains elements of the input vector. For each element e, the back of the deque is removed if it is strictly smaller than e. Then e is added on the back of the deque and elements that are out of the current window are removed. The minimum of the current window is then the front of the deque. Time: $\mathcal{O}(|v|)$, but requires time consuming memory allocations.
- "*fixed windows*": the approach described in Section 2.4, but allocating new vectors. Time: $\mathcal{O}(|v|)$, but requires time consuming memory heap allocations.
- Our proposal: "***fixed windows in place***": based on the fixed approach we also introduced (previous item), but without heap allocation, as described in algorithm 2. Time: $\mathcal{O}(|v|)$.

We propose various tests, highlighting the behaviors of the presented algorithms in different contexts: v is made up of random integers with either a small (size 9) or a large (size 100) window, and v is made up of increasing integers. In all cases, our proposal is the fastest algorithm. Recall that the implementation, of independent interest, is available at `https://github.com/lrobidou/sliding-minimum-windows` along with a recipe to reproduce and extend this benchmark.

As shown in Fig. 1, when $size\_window$ is set to 9, approaches can be divided into two groups: those with heap allocations and those without. The fixed window approach with heap allocations is slower than keeping the last minimum ($\mathcal{O}(size\_window \times |v|)$ at worst), but once heap allocation is prevented (our proposal), this outperforms any other implementation, including keeping the last minimum by a factor 2. Heap allocations are costly: the differences between approaches in $\mathcal{O}(size\_window \times |v|)$ with heap allocation and in $\mathcal{O}(|v|)$ without them are negligible in practice (as long as $|v|$ is small enough, see Fig. 2).

Results presented Figure 2 show that the naive approach in $\mathcal{O}(size\_window \times |v|)$ is significantly slower than other approaches, even if it does not require heap allocation.

Keeping a reference to the minimum from the previous window is simpler than using a fixed window but it is two times slower. Moreover, if the input vector contains increasing values, it

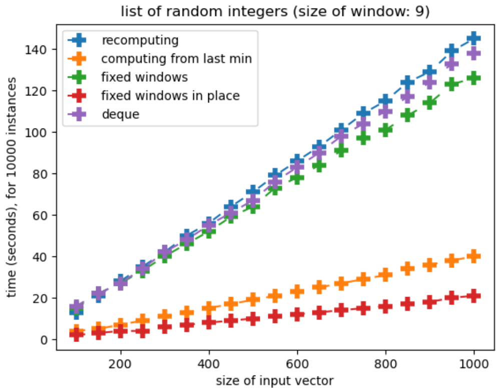

Figure 1: Comparison of five approaches for computing sliding window minimum. The input vector is composed of random integers. The size of the window is set to 9 and for each size of the input vector, 10000 instances were tested.

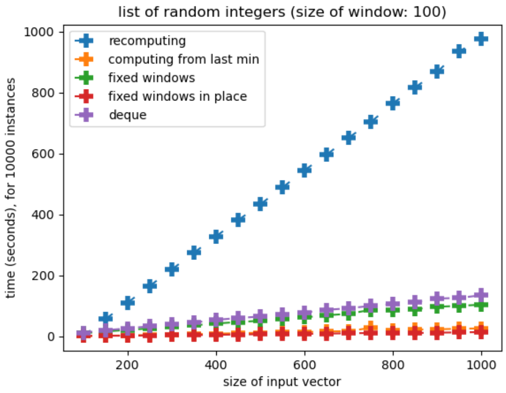

Figure 2: Comparison between 5 approaches for computing sliding window minimum. The input vector is composed of random integers. The size of the window is set to 100.

requires recomputing every window. Indeed, in this case, the minimum from any window is its leftmost element, and as it is out of the next window, the next window needs to be recomputed. See Fig. 3, to see the impact on run time. As illustrated in the figure, the strategy of keeping a reference to the minimum of each window is slower than when applied to random values. In any case, the query time of the method we propose is constant regardless of the input vector's content.

3.2 Comparing `fimpera` and a counting Bloom filter with multiple hash functions

As stated in the main manuscript, Section 2.2.1, the `fimpera` strategy may seem similar to associating multiple hash functions to a k-mer through its constituent s-mers, albeit two consecutive k-mers share z s-mers, allowing to not query the same s-mer multiple times.

In this section, we compared the reported abundance when indexing k-mers through $z+1$ s-mers compared to indexing k-mers through $z+1$ independent hash functions.

Using the overestimation score defined in Section 3.4 of the main manuscript, it is possible to compare `fimpera` ($z = 3$) using one hash function (ie. using 4 s-mers per k-mers) and using 4 independent hash functions. Using the same memory budget, $z = 3$ allows attaining an overesti-

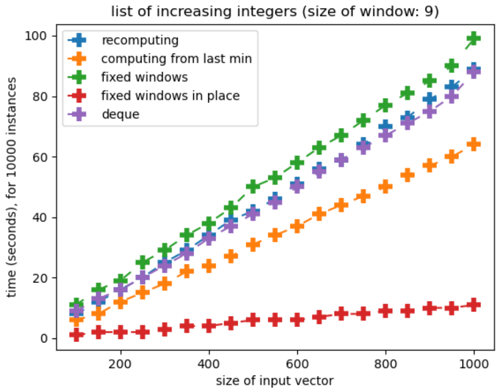

Figure 3: Comparison between 5 approaches for computing sliding window minimum. The size of the window is set to 9 and the input consists of an array of increasing values.

mation score of 98169 while using 4 independent hash functions allows reaching an overestimation score of 57967168 (the lower the better). The false positive rate attained by using $z = 3$ is $\approx 0.36\%$ while using 4 independent hash functions leads to a drastic increase of the false positive rate to 99.94%. This is due to the fact that using 4 independent hash functions saturates the filter and most absent k-mers calls lead to false positives.

3.3 Comparison between s_{ab} and abundance of s-mers

As stated in Section 2.2.2 of the main manuscript, we index the s_{ab} of each s-mer instead of its abundance in the input dataset. This is motivated by the fact that the s_{ab} of a s-mer α is lower or equal [1] to the abundance of α in the input dataset. For instance, consider a s-mer α that occurs in two k-mers respectively with an abundance of one and two. Then, the abundance of α is three $(= 1 + 2)$, while the s_{ab} of α is two $(= \max(1, 2))$. Storing the s_{ab} of α instead of its abundance lowers the abundance overestimations, as it avoids accumulating the abundances of distinct k-mers it belongs to.

However, computing the s_{ab} is time-consuming, as it requires parsing all s-mers given an input composed of counted k-mers. Thus, keeping compatibility with most k-mer counter requires either that the indexation step is performed by `fimpera`, or the use of the abundance of s-mers instead of the s_{ab}.

To measure overestimations achieved when indexing the abundance of s-mers instead of their s_{ab}, we provide additional results (same datasets as those used in the main manuscript). In the main manuscript, we indexed the $\lfloor \log_2 \rfloor$ values of abundance for limiting the size of the `cBF`. In this section, we show overestimation results computed on exact abundances stored using 8 bits, and thus bounded to 255. We used $k = 31$ and $s = 28$. This is motivated by the fact that, else, no difference could be seen.

In Fig. 4, we show that overestimations introduced by the use of the abundance of each s-mer instead of s_{ab} are limited. This can be numerically estimated:

- the overestimation score comparing a raw counting AMQ to the ground truth is $\approx 8.6 \times 10^7$;
- the overestimation score comparing `fimpera` to the ground truth is $\approx 10^4$ (three order of magnitude smaller);
- the additional overestimation score comparing the indexing of the abundance of s-mers instead of their $s - abundance$ with `fimpera` is $\approx 2 \times 10^4$ which appears negligible.

[1] except in some extremely rare cases with palindromic s-mers as described below

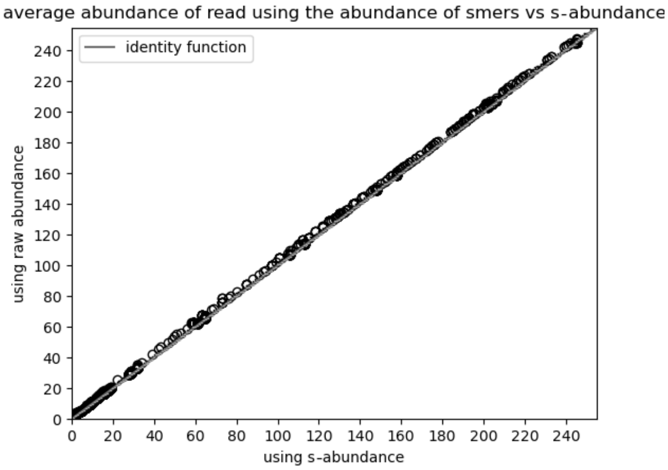

Figure 4: Comparison between the average abundance of k-mers from reads query results.

Extremely counter-intuitive case: the abundance of a s-mer can be smaller than its s_{ab}

Actually, in some rare setups, the abundance of s-mer can be smaller than its s_{ab}, and even lower than the abundance of any k-mer that contains it (which would lead to an underestimation). This is only possible when indexing and querying canonical k-mers. This case happens with s even, when two k-mers having the same canonical form overlap over s characters. On a sequence of size $k+1$ composed of such overlap of two k-mers having the same canonical form, their canonical abundance in this sequence is 2, whereas the s-mer being the suffix of the first k-mer and the prefix of the second has an abundance of 1, thus smaller than its $s_{ab} = max(2) = 2$.

For instance, consider the sequence $seq = TACGTA$ and $k = 5, s = 4$. The first k-mer is $TACGT$ and the second k-mer is $ACGTA$. Both have the same canonical form $ACGTA$, whose abundance is then equal to two. The s-mer they share, $ACGT$, exists only once in seq, so its abundance is one, lower than its s_{ab} equal to two as provided by the abundance of the unique (in this situation) abundance of the canonical k-mer it belongs to.

This setup is rare (541 out of 281032928 s-mers of the indexed TARA dataset, i.e.: 0.000193 % of its s-mers). In practice, this led to zero underestimation of the average of any read. Even if using the abundance of s-mers instead of their s_{ab}, and using their canonical versions breaks the theoretical absence of underestimation, we consider the biological impact of these underestimations as perfectly negligible.

3.4 Effects of choosing the k value at query time

As shown in the main manuscript in Table 2, the false positive rate of `fimpera` drops with regard to z when indexing k-mers through their constituent s-mers. However it can be useful to index s-mers, and then choose k at query time. This allows the user to choose any k value ($\geq s$) even after the indexation step. The major difference of this approach with respect to having s and k fixed at indexing time is that, since k is unknown when indexing, we cannot compute the s-abundance of s-mers. This is due to the fact that computing the s-abundance of s-mers requires knowing the k value. Thus, in this setup, we rely on the abundance of s-mers instead of their s-abundance.

In Table 1, we show that the false positive rate drops with regard to z, with a fixed s value.

As a side note, as previously mentioned, when choosing k at query time, one cannot compute the s-abundance of the s-mers. Hence, in this setup, the abundance of s-mers is higher than the situation when k and z are known at indexing time, in which one would have used the s-abundance.

Consequently, less s-mers have an abundance lower than the threshold and are filtered out, slightly increasing the FP rate, comparatively with results presented in the main manuscript.

Note that choosing z at query time allows using a high value, such as 35, while still limiting the construction false positives. This is due to the fact that construction false positive rate mainly depends on the s value.

**z**	0	1	2	3	4	5	6	7	8	35
False positive rate (%)	34.972	12.236	4.286	1.507	0.536	0.197	0.079	0.039	0.025	0.031
Of which: construction FP (%)	0	0.007	0.036	0.159	0.584	2.126	6.546	15.768	28.236	89.269
Incorrect abundance calls (%)	2.786	1.362	2.095	3.248	4.542	5.803	7.122	8.535	9.972	55.495
Of which: constr. overest. (%)	0	65.114	83.518	90.079	93.296	94.751	95.644	96.290	96.841	99.692

Table 1: Influence of the z parameter on the quality of the results when s if fixed. "*constr.*" stands for "*construction*" and "*overest.*" stands for "*overestimation*". The Incorrect abundance calls are computed over true positive calls only.

3.5 Effect of not grouping values using log functions

In the main manuscript, Section 2.5, we indexed the $\lfloor \log_2 \rfloor$ values of abundance for limiting the size of the `cBF`. In this section, we show overestimation results computed on exact abundances stored using 8 bits, and thus bounded to 255. We used $k = 31$ and $s = 28$.

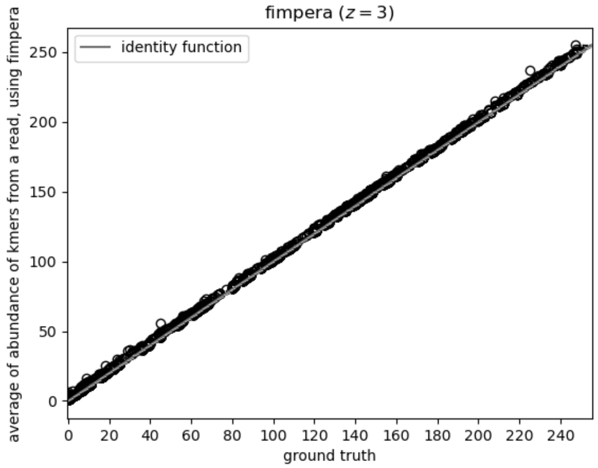

Figure 5: Average abundance of reads reported by `fimpera` ($k = 31, s = 28$) using one hash function with regard to the ground truth, compared to the identity function.

Fig. 5 shows the response of `fimpera` for each ground truth abundance, not considering abundances as their $\lfloor \log_2 \rfloor$ values. The median of response for each ground truth value is close to that value (most box plots consist in the first quartile, the median and the third quartile being equal to the ground truth). Using the same overestimation score as in Section 3.4 (main manuscript), we compared `fimpera` ($z = 3$) and the underlying counting Bloom Filter. Using the same memory budget, `fimpera` has an overestimation score of 98169 while the counting Bloom Filter has an overestimation score of 85873897 (3 orders of magnitude higher, the lower the better).
Thus, the results of `fimpera` still hold when not using any surjective function to group values, eg. by their log.
